# Persuading Patients Using Rhetoric to Improve Artificial Intelligence Adoption: Experimental Study

**DOI:** 10.2196/41430

**Published:** 2023-03-13

**Authors:** Glorin Sebastian, Amrita George, George Jackson Jr

**Affiliations:** 1 Georgia Institute of Technology Atlanta, GA United States; 2 Georgia State University Atlanta, GA United States; 3 Capella University Minneapolis, MN United States

**Keywords:** communication strategies, artificial intelligence adoption, AI adoption, privacy concerns, trust, technology acceptance, health IT

## Abstract

**Background:**

Artificial intelligence (AI) can transform health care processes with its increasing ability to translate complex structured and unstructured data into actionable clinical decisions. Although it has been established that AI is much more efficient than a clinician, the adoption rate has been slower in health care. Prior studies have pointed out that the lack of trust in AI, privacy concerns, degrees of customer innovativeness, and perceived novelty value influence AI adoption. With the promotion of AI products to patients, the role of rhetoric in influencing these factors has received scant attention.

**Objective:**

The main objective of this study was to examine whether communication strategies (ethos, pathos, and logos) are more successful in overcoming factors that hinder AI product adoption among patients.

**Methods:**

We conducted experiments in which we manipulated the communication strategy (ethos, pathos, and logos) in promotional ads for an AI product. We collected responses from 150 participants using Amazon Mechanical Turk. Participants were randomly exposed to a specific rhetoric-based advertisement during the experiments.

**Results:**

Our results indicate that using communication strategies to promote an AI product affects users’ trust, customer innovativeness, and perceived novelty value, leading to improved product adoption. Pathos-laden promotions improve AI product adoption by nudging users’ trust (n=52; *β*=.532; P<.001) and perceived novelty value of the product (n=52; *β*=.517; P=.001). Similarly, ethos-laden promotions improve AI product adoption by nudging customer innovativeness (n=50; *β*=.465; P<.001). In addition, logos-laden promotions improve AI product adoption by alleviating trust issues (n=48; *β*=.657; P<.001).

**Conclusions:**

Promoting AI products to patients using rhetoric-based advertisements can help overcome factors that hinder AI adoption by assuaging user concerns about using a new AI agent in their care process.

## Introduction

### Background

Artificial intelligence (AI) technologies refer to any device that perceives its environment and takes action to maximize its chance of success [1]. Some examples of these technologies include machine learning, rule-based systems, natural language processing, and speech recognition. Technological advancements and improved computing capabilities have increased the proliferation of AI products. The adoption of AI products in health care has the potential to transform the health care industry, with medical AI forecasted to exceed a market size of ≥US $30 billion by 2025 [2].

Prior research on technology innovation has used the Technology Acceptance Model (TAM), Theory of Planned Behavior, and Unified Theory of Acceptance and Use of Technology to examine AI adoption and use [3]. The Unified Theory of Acceptance and Use of Technology has been widely used to explain intentions for technology use in various fields, including intelligent health care systems [4,5], whereas the TAM discusses the impact of perceived usefulness and ease of use on the behavioral intention to use and the actual use of technology. In addition, the TAM has been extended to derive the Value-based Adoption Model [6] to study the influence of benefits, such as usefulness and enjoyment, and sacrifices (such as technicality and perceived fee) on the perceived value of technology adoption. Moreover, AI adoption research has examined the antecedents of AI adoption [7]. For example, studies have established the influence of antecedents such as perceived usefulness, consumer innovativeness, and reference groups on the adoption intention of wearable health care technology [8]. Prior research points to the presence of antecedent hindering factors to AI adoption, such as privacy concerns, lack of trust (especially related to the accuracy, efficiency, and precision of AI), different degrees of consumer innovativeness, and lack of perceived novelty value [7,9]. Although prior research has established that communication strategies can persuade users to overcome concerns about using technology [10], to the best of our knowledge, this has not been empirically validated or studied in the context of AI product technology adoption. Addressing this research gap would be a substantial contribution, because communications have been found to be persuasive in health care outreach programs [11]. Therefore, different communication strategies can be used to persuade users to adopt AI products, particularly in health care.

The Art of Rhetoric by Aristotle [12], written in the 4th century BC, is highly regarded as a seminal work in argumentation and persuasion that forms the basis for communication strategies. In his work, Aristotle describes 3 main methods of persuasion: logos (logical), ethos (ethics), and pathos (emotion). Logos uses logical reasoning and evidence for persuasion. Ethos uses character, credibility, ethics, and previous persuasion achievements. Pathos uses emotions and passion for persuasion. Furthermore, in The Art of Rhetoric, Aristotle defines the 3 styles of oration: deliberative (political), forensic (legal), and epideictic (ceremonial) [13]. With these foundational principles established, Aristotle describes how logos, ethos, and pathos may be successfully applied in different forms of oration, that is, differing forms of messaging and communication. Knowing how and when to apply logos, ethos, and pathos in a persuasive argument allows a speaker to pattern their rhetorical style to best suit an intended audience. Organizations aiming to increase the adoption of their AI products can also choose appropriate communication strategies to market the product to the intended audience. In this study, we aimed to understand the communication strategies that can aid in overcoming user concerns, thereby improving technology adoption, specifically AI adoption. Therefore, the research question we sought to answer was as follows: *Do communication strategies assuage barriers to AI adoption?*

Research on technology adoption and communication strategies suggests that managers of technology-based products could use communication strategies that promote the use of their technologies [10]. This study attempts to extend this finding to AI adoption in health care by studying the influence of communication strategies to assuage hindrances in AI adoption from a patient’s perspective. We conducted experiments with potential AI product users to understand their intention to adopt an AI product when various communication strategies were used. The results of our study have major implications for both theory and practice. In terms of theoretical contributions, we have identified how using the right communication strategies could alleviate hindering factors identified in the technology adoption literature, such as privacy concerns, trust, and perceived novelty, which in turn can influence the behavioral intention to use and actual system use (specifically AI products in health care). Our study also makes a substantial practical contribution by making both health care AI product manufacturers and clinicians aware of structuring their communication with patients to persuade them to adopt the AI product that can be beneficial to the practitioner and patient.

### Relevant Literature

For the literature review, we did a Boolean search using keywords such as “Technology Acceptance Model” AND “communication strategies (ethos, pathos, logos)” as well as “antecedents” OR “hindrances” OR “inhibitor” OR “enabler” AND “AI adoption” OR “technology adoption” specifically in health care to find relevant studies in top information systems, health informatics, and computer science journals (eg, *Computers in Human Behavior*, *Management Science*, *Journal of Medical Internet Research*, *Information Systems Journal*, and *MIS Quarterly*). We found that prior studies summarize the enablers and inhibitors in AI adoption from consumer and practitioner perspectives (Table S1 in Multimedia Appendix 1 [7-11,14-22]), how these factors influence behavioral intention to use, and the actual system use and ultimately impact technology adoption.

### Factors Influencing AI Adoption

#### Overview

AI is defined as the intelligence demonstrated by machines, in contrast to the natural intelligence displayed by humans and other animals. However, in the present context of medical imaging, a more specific definition may be more appropriate: “a system’s ability to correctly interpret external data, to learn from such data, and to use what was learned to achieve specific goals and tasks through flexible adaptation” [23]. AI research has examined the key challenges and hindrances to AI adoption and how these challenges can be overcome [24].

Some of the hindering factors identified included privacy concerns, trust (especially related to the accuracy, efficiency, and precision of health IT systems), consumer innovativeness, and perceived novelty value (Tables S1 and S5 in Multimedia Appendix 1). The implications of these factors on AI adoption are discussed in subsequent sections.

#### Privacy Concerns

Data privacy concerns can hinder technology adoption, particularly when many data privacy directives, such as the Health Insurance Portability and Accountability Act of 1996, create national standards to protect sensitive patient health information. Similarly, widespread news reports of data breaches can trigger a user’s privacy concerns. In such situations, users could have concerns with sharing sensitive personal information (eg, medical information) with AI bots or applications. In addition, data may need to be shared across multiple institutions and geographies (eg, telehealth), which might also raise one’s privacy concerns (Table S5 in Multimedia Appendix 1). Research also notes that lack of data integrity and continuity and lack of standards for data collection, format, and quality are some of the concerns impacting stakeholders in the adoption of AI in public health care [24]. In addition, most health care professionals, who are obligated to promote the tenets of confidentiality, do not understand their respective responsibilities toward medical confidentiality [25]. Security and privacy concerns influence both technology trust and user well-being, as well as the behavioral intention to use AI products [14], including consumers’ intention to adopt wearable health care technology [8].

#### Trust

Trust is a psychological state broadly defined based on 3 main dimensions, namely, benevolence, integrity, and ability. Prior research on AI adoption has established that technology trust influenced the behavioral intention of the use of AI products [14]. Trust is the cornerstone of effective AI user interactions, such that it affects how much users rely on AI [26]. Many users are skeptical about using technologies, such as AI assistants, owing to certain perceived risks. For example, many patients trust a surgeon more than a robotic surgical system, despite these systems being as efficient as a surgeon [15]. Furthermore, many aspects of machine learning, such as deep learning, remain a black box, with the lack of explainability and transparency impacting the trust-building process [27]. Trust in AI can also be influenced by several personal factors, such as education, past experiences, user biases, and emotions, as well as properties of the AI system, including controllability, model complexity, embedded biases, and reliability (ie, whether AI technology can perform a task predictably and consistently) [28].

#### Consumer Innovativeness

Customer innovativeness is the potential of consumers in a target segment to adopt a new product or technology [29]. Consumer research shows that people are likely to adhere to their existing routines, characterized by risk aversion and a general preference to buy familiar products [30]. The users who are ready to buy or try a product as soon as it hits the markets are considered consumer innovators and, in most cases, are opinion leaders or influencers. Prior research points out that user innovativeness can influence the behavioral intention to use AI products [14], including the adoption intention of wearable health care technology [8]. Consumer innovativeness plays a critical role in AI adoption and can be influenced by marketing campaigns from the organization [31], especially in the case of the adoption of medical technologies [32,33].

#### Novelty Value

Novelty value is the value characteristic that users obtain from using or adopting a new product, service, or technology that is surprising and fresh [34]. The conceptualization of innovativeness by Hirschman [34] focuses on consumer desires to obtain information about innovations that aid in the adoption or use of novel products and technology. Innovativeness is equated with inherent novelty seeking and is defined as “the desire to seek out the new and different.” [34] The perceived usefulness of a novel product also drives its increased adoption [16,35]. Furthermore, research notes that novelty (epistemic) values and emotional and social values significantly influence the adoption of new technologies [17]. Although these factors can hinder AI adoption, research on technology adoption suggests that managers of technology-based products could use communication strategies that promote the use of their technologies. Prior research indicates that communication strategies can persuade users to overcome concerns about using technology [10].

### Communication Strategies in Technology Adoption

Prior studies have examined the impact of communication strategies on influencing consumer behavior in technology adoption (Table S1 in Multimedia Appendix 1). Knowing how and when to apply logos, ethos, and pathos in a persuasive argument allows a speaker to pattern their rhetorical style in a manner that best suits an intended audience. Research on technology adoption and communication strategies notes that communication strategies used by managers of technology-based products to promote the use of their technologies have an impact on technology adoption [10]. However, the impact of communication strategies on overcoming the hindering factors in AI adoption has received scant attention. Innovative technologies such as AI that promise enormous improvements in processes, goal attainment, outcomes, safety, etc, have many uncertainties (eg, lack of trust or confidence) that can impact adoption. AI product creators can aim at overcoming these barriers through active marketing campaigns with messages tailored to reach the right audience. Similar to other areas, communication strategies can be used to overcome risks. For example, Wieder [11] proposed a theoretical approach to using communication strategies to deliver a persuasive message on communicating radiation risk. Pathos communication justifications impact emotions and are likely to elicit powerful yet unsustainable social actions [18]. Logos approaches appeal to the logical part of the mind; they tend to elicit methodical calculation of means and ends to achieve efficiency or effectiveness [18]. Ethos justifications impact moral or ethical sensibilities [18]. A sequence of justifications starting with pathos and logos produces pragmatic legitimacy, whereas ethos would generate moral legitimacy [18]. For example, rhetorical modes of logos (rational) and pathos (emotional) were used to change the UK’s societal attitudes toward sharing health data [19]. In addition, the presence of both pragmatic and moral justification is required to create cognitive legitimacy [18].

### Research Model and Hypotheses

**Figure 1:**
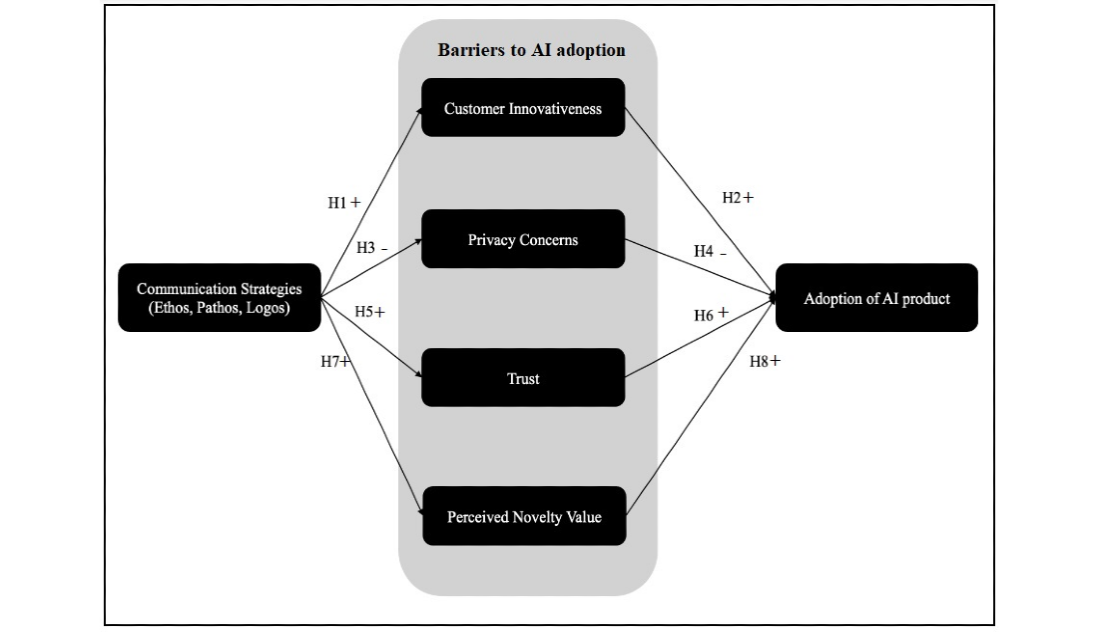
Research model. AI: artificial intelligence; H: hypothesis.

Researchers have investigated various factors influencing technology adoption, such as customer innovativeness, privacy concerns, trust, and novelty value. Specifically, in the context of AI adoption, research has studied the impact of privacy concerns, technology trust, and consumer innovativeness, positively impacting behavioral intention to use the technology [8,14]. However, the effect of communication strategies on assuaging barriers to AI technology adoption has received limited attention. To address this research gap, we propose the research model in Figure 1 to hypothesize that communication strategies impact various concerns and traits of end users (patients), thereby indirectly influencing the adoption of AI technology. The literature has already considered the influence of these barriers on perceived ease of use or usefulness and, in turn, intention to use. We have not included perceived ease of use and perceived usefulness in the model for parsimony.

Consumer innovativeness, “the degree to which an individual is earlier in adopting new ideas than the average number of his or her social system” [36], includes creativity and adaptability to change. Consumer innovativeness is highly acknowledged by marketers for the successful diffusion of innovation to make businesses more profitable and competitive [36].

Ethos, a communication strategy that uses credibility and ethics for persuasion, can mitigate any risk perceived with using the AI product. For example, American Medical Association–endorsed products will be viewed as meeting certain standards and regulations, which alleviates concerns related to safety or ethics or errors when using the AI product. Similarly, Food and Drug Association–approved AI and machine learning medical devices will be viewed favorably by the users, including the innovative customers. It has been established that ethos positively affects consumer innovativeness [37]. An endorsement by a trusted figure improves consumer confidence in the product, thereby increasing adoption [38].

Pathos, a communication strategy that uses emotions and passion for persuasion, can alleviate emotional concerns when using the AI product. For example, a happy customer endorsing an AI product can influence the user’s perception of the product, thereby viewing the product as something that will improve their quality of life [39]. When someone similar to us endorses a product with a happy emotion, it improves innate consumer innovativeness [40]. consumer’s innovativeness can be nudged by their passion to be unique [36]. The need to be unique is nudged when the advertisement demonstrates a unique opportunity, thereby positively influencing consumer innovativeness [41].

Logos, a communication strategy that uses logical reasoning and evidence for persuasion, can provide fact-based evidence to alleviate perceived risks. It provides customers reassurance based on historical successes, which in turn improves confidence in the product. Providing logos-based information on product credibility furthers consumer confidence in the product and improves the adoption rate. For example, when a physician advises a patient on a course of treatment, the physician will present relevant medical evidence and explain why the benefits derived from the recommended course of action will likely provide the best outcome for the patient while outweighing the potential risks. Logic-based arguments such as these are designed not only to inform but also to influence and persuade patient behavior.

Studies have established that consumer innovativeness influences the behavioral intention to use AI products [14] and consumers’ adoption intention for wearable health care technology [8]. Studies found that when customer innovativeness is high, users are more likely to accept the innovative technology [42]. With AI being a new technology in health care, nudging customer innovation can improve acceptance of the AI product. For example, e-learning and web-based classes are still in the developmental phase in many parts of the world. Sharing the positive effects of e-learning, including performance expectancy and social influence, has helped spark customer innovativeness and interest in offerings that lead to increased adoption [43,44]. Therefore, the following were our hypotheses (Hs):

- H1: using communication strategies for promoting the AI product can positively impact customer innovativeness.
- H2: nudging customer innovativeness can improve acceptance of the AI product being promoted.

Prior privacy concerns, computer anxiety, perceived control, and app permission concerns can affect a user’s privacy concerns when using an AI product [45]. Studies have established that privacy concerns influence the behavioral intention to use AI products [14], including consumers’ intention to adopt wearable health care technology [8].

Advertisements using an ethos communication strategy will help alleviate ethical concerns related to compliance, standards of data collection, format, preservation of data integrity, and data integration and continuity. For example, an ad in which a celebrity endorses an AI product and clearly mentions that it is safe to use and compliant with most data directives would alleviate patients’ privacy concerns. By contrast, advertisements using a pathos communication strategy will aid in reconciling the cognitive dissonance that a user may have about using the product. For example, an ad that showcases an older adult couple being taken care of by a humanoid robot can emotionally persuade the end user and reduce any concerns the user could have regarding AI. The cognitive dissonance may create an unpleasant emotional state that can be alleviated through an appropriate pathos communication strategy. Finally, when advertisements use logos communication strategy, it can provide evidence of adherence to privacy policies (ie, notice, enforcement, access, security, or choice) that can alleviate privacy concerns [46]. For example, providing assurance that the product is compliant with various data privacy directives, such as the Health Insurance Portability and Accountability Act of 1996 and General Data Protection Regulation, along with clarifying what this compliance entails, helps address the concerns identified in research [45].

Previous studies have found that when the perceived security of a specific technology is high, users are more likely to accept innovative technology [42]. This justifies our hypotheses that privacy and security are barriers preventing the adoption of AI. For example, recently, social media technology giants such as Google have started advertisements to disseminate information on privacy considerations while designing their products, which is clearly aimed at users addressing their privacy concerns, thereby nudging them to further use their products. Therefore, we hypothesized the following:

- H3: using communication strategies for promoting the AI product can alleviate privacy concerns with using the product.
- H4: alleviating privacy concerns can improve acceptance of the AI product being promoted.

Trust, a psychological construct that encompasses an emotional and a logical aspect [47], can influence a user’s perception of using an AI product. When an AI product is advertised using an ethos communication strategy, the logical aspect of trust can be nudged. For example, when a credible organization such as the American Medical Association promotes the implementation of AI in health care and talks about the benefits of AI adoption, it positively influences many people by alleviating their concerns about AI, thereby enhancing users’ trust. Similarly, when the AI product is marketed using a pathos communication strategy, the emotional facet of trust can be nudged. For example, AI used for medical procedures enables better precision and higher success of these procedures, thereby allowing the best care for patients. It can be emotional for users to see their family getting the best possible care, and this emotional impetus allows them to trust AI better. Finally, marketing an AI product by using a logos communication strategy can improve the logical component of trust. For example, listing the benefits of automation in health care, such as accuracy and efficiency, including the hours and effort saved, helps build trust in AI products.

Prior research observed trust as an important antecedent of technology acceptance [48] and behavioral intention to use AI products [14]. The authors point out that trust provides a measurement of the subjective guarantee that the agent can make good on its side of the deal, behave as promised, and genuinely care [48]. With AI being a new technology in health care, where users are uncertain of the risks posed by using the technology, nudging the emotional and logical facets of trust can improve acceptance of the AI product. Therefore, we hypothesized the following:

- H5: using communication strategies for promoting the AI product can improve trust in using the product.
- H6: enhancing trust can lead to better acceptance of the AI product being promoted.

The novelty of the content or novelty value of a new technology will positively influence (1) its perceived ease of use and (2) its perceived usefulness [49,50]. Communication strategies such as pathos, ethos, and logos can improve the perceived ease of use and perceived usefulness of a new technology-laden product, such as AI products, because it communicates the novelty value of the product. When using pathos messaging in the marketing of AI products, marketers are convincing users of the product’s novelty through the manipulation of emotions, which in turn improves the product’s perceived usefulness and ease of use. For example, marketing Pria (an AI product) by stating that the product is easy to use by an older adult can convince consumers about the automated medicine dispenser and its ease of use and usefulness for someone near and dear to them. Similarly, when using ethos messaging in the marketing of AI products, marketers are convincing users of a product’s novelty through advocacy from a credible source, which in turn improves the product’s perceived usefulness and ease of use. For example, marketing Pria (an AI product) using celebrities or agencies such as the American Medical Association can convince consumers about the automated medicine dispenser and its ease of use and usefulness for themselves. Similarly, when using logos messaging in the marketing of AI products, marketers are convincing users of the product’s novelty through facts and evidence, which in turn improves the product’s perceived usefulness and ease of use. For example, marketing Pria (an AI product) by showing statistics about improvements in medicine adherence can convince consumers about the automated medicine dispenser and its ease of use and usefulness for themselves. Improved perceived ease of use and usefulness can positively impact technology adoption [16,35], which also applies in the context of novel technology [50], including AI products. Therefore, we hypothesized the following:

- H7: using communication strategies for promoting the AI product can positively influence the perceived novelty value of the product.
- H8: perceived novelty value can lead to better acceptance of the AI product being promoted.

## Methods

### Experiment Design

To test the hypothesis, we conducted 4 experiments in which we manipulated the communication strategy (ethos, pathos, and logos) using screenshots of advertisements for a product (Figures S1-3 in Multimedia Appendix 1). The participants were randomly assigned to each group. A control group was also included in the experiment to ensure the primes worked. Our primes were designed to ensure that communication strategies (ethos, pathos, and logos) were induced (Figures S1-3 in Multimedia Appendix 1). Participants were asked advertisement effectiveness assessment questions as a manipulation check to identify whether the primes induced different responses (Table S2 in Multimedia Appendix 1). We followed up on each experiment by asking questions to evaluate trust, novelty value, customer innovativeness, privacy concerns, and AI adoption of the product for which the advertisement was shown. We adapted scales from the literature to measure trust [51,52], novelty value [51,53], customer innovativeness [54], privacy concerns [55], and AI adoption [56] (Table S3 in Multimedia Appendix 1). The items measuring trust, novelty value, customer innovativeness, privacy concerns, and AI adoption ranged from 1 (strongly disagree) to 5 (strongly agree).

We collected data via Amazon Mechanical Turk (AMT), where we recruited AMT users with a Human Intelligence Task approval rate greater than 95%. One of the main advantages of using the AMT population is that it improves the generalizability of inferences as compared with traditional data collection methods [57]. The AMT workers received a small monetary reward for their participation.

### Manipulation Check

We assessed whether the manipulation was successful. The participants were asked to rate questions related to the effectiveness of each advertisement. For example, participants were asked to rate “this advertisement was relevant/meaningful/important to me” (“*1 strongly disagree*...*5 strongly agree*”; see Table S4 in Multimedia Appendix 1 for the entire list). An ANOVA was conducted and a significant mean difference between the communication strategy conditions indicated that the manipulation was successful (Table 1). Participants in the pathological condition of the treatment group reported scores (mean 3.37, SD 0.90) different from those in the ethos condition (mean 3.52, SD 0.97) and logos condition (mean 3.43, SD 0.89). Thus, the results confirmed the effectiveness of communication strategy manipulation.

**Table 1:** Mean score and ANOVA results for induced communication strategy (CS) conditions.

Experimental condition and CS type		Sample size, n	Values, mean (SD)	ANOVA	
**CS**					*F*_1,144_=43.79; P<.001
	Pathos	52	3.37 (0.90)		
	Logos	48	3.43 (0.89)		
	Ethos	50	3.52 (0.97)		

### Sample Characteristics and Psychometrics

We restricted the sample to AMT workers. Table S6 in Multimedia Appendix 1 presents the descriptive statistics. Partial least squares (PLS) analysis using SmartPLS was used to validate the psychometric properties of our measures and test the paths hypothesized in Figure 1. We chose PLS because it permits the modeling of latent variables and the simultaneous assessment of the measurement and structural models, while placing minimal demands on sample size and distributional assumptions [58,59]. We first examined the psychometric properties of our measures using the measurement model and then tested our hypotheses using a structural model.

We assessed the reliability and validity of our measurement items by examining the factor loadings, Cronbach α, and average variance extracted. The results of our analyses indicate that the scales had good reliability and validity (Tables S7-12 in Multimedia Appendix 1). We then conducted single-factor test by Harmon [60,61] to rule out common method bias. The results suggest that common method bias is unlikely to be a significant problem in our data given that more than one factor emerged from the factor analysis as well as the fact that the first factor did not account for most of the variance in our data (Tables S7-12 in Multimedia Appendix 1).

### Ethics Approval

Data collection proceeded after obtaining approval from the institutional review board. The review board provided permission to proceed in its determination letter issued on November 18, 2021 (request # HR-4022). All participants were required to provide informed consent to participate in the study at the beginning of the web-based questionnaire. Data were handled in accordance with US regulations.

## Results

### Overview

To test our hypotheses, we estimate 3 PLS models for each communication strategy. Model 1 examined the effect of the path communication strategy for a patient AI product on the dependent variable (ie, AI adoption). Model 2 tested the influence of an ethos communication strategy for a patient’s AI product on AI adoption. Model 3 tested the influence of the logos’ communication strategy for a patient AI product on AI adoption. Table 2 presents the results of the 3 models. To test H1 to H8, we assessed the structural model by examining the path coefficients and their significance levels for each model. The path coefficients were computed for each group. The significance levels for the effects were computed in SmartPLS using 1000 bootstrap samples [61].

**Table 2:** Results of 3 models of communication strategy (CS)^a^.

PLS^b^ path	CS					
	Pathos	P value	Ethos	P value	Logos	P value
CS→AI^c^ adoption	−0.145	.38	0.199	.14	0.227	.09
CS→trust	0.532	<.001	0.662	<.001	0.657	<.001
Trust→AI adoption	0.361	.01	0.140	.35	0.474	.008
CS→privacy concerns	−0.332	.01	0.106	.52	−0.267	.05
Privacy concerns→AI adoption	−0.111	.34	0.105	.28	−0.074	.49
CS→customer innovativeness	0.514	<.001	0.465	<.001	0.602	<.001
Customer innovativeness→AI adoption	−0.121	.45	0.417	<.001	−0.017	.91
CS→novelty value	0.517	.001	0.666	<.001	0.582	<.001
Novelty value→AI adoption	0.591	<.001	0.199	.25	0.104	.61

^a^Pathos: *R^2^*=0.584, adjusted *R^2^*=0.539; ethos: *R^2^*=0.614, adjusted *R^2^*=0.570; logos: *R^2^*=0.555, adjusted *R^2^*=0.502.

^b^PLS: partial least squares.

^c^AI: artificial intelligence.

### Hypothesis Testing

We ran a regression model with communication strategy as the independent variable; customer innovativeness, trust, perceived novelty value, and privacy concern as mediators; and AI adoption as the dependent variable for the treatment groups.

For the pathos condition, the communication strategy predicted trust and perceived novelty value. The results showed that the coefficient of communication strategy on trust was positive (*β*=.532; P<.001) and that the coefficient of communication strategy on perceived novelty value was positive (*β*=.517; P=.001). The coefficient of trust in AI adoption was also positive and significant (*β*=.361; P=.01). In addition, the coefficient of perceived novelty value on AI adoption was positive and significant (*β*=.591; P<.001). While there was an effect of communication strategy on privacy concerns (*β*=−.332; P=.01) and customer innovativeness (*β*=.514; P<.001), the effect of privacy concerns (*β*=−.11; P=.34) and customer innovativeness (*β*=−.12; P=.45) on AI adoption was insignificant, but the magnitude confirmed previous theoretical findings that these factors inhibited AI adoption. Whereas H4 and H8 were not supported, our results indicated support for H1, H2, H3, H5, H6, and H7 in the pathos condition.

For the ethos condition, communication strategy predicted customer innovativeness. The results showed that the coefficient of communication strategy on customer innovativeness was positive (*β*=.465; P<.001). The coefficient of customer innovativeness on AI adoption is also positive and significant (*β*=.417; P<.001). The coefficients of communication strategy on perceived novelty value (*β*=.666; P<.001) and trust (*β*=.662; P<.001) were positive and significant. However, the coefficients of perceived novelty value (*β*=.199; P=.25) and trust (*β*=.140; P=.35) in AI adoption were insignificant. The effect of communication strategy on privacy concerns was also insignificant (*β*=.106; P=.52), as was the effect of privacy concerns on AI adoption (*β*=.105; P=.28), but the magnitude confirmed previous theoretical findings that privacy concerns inhibited AI adoption. Whereas H1 and H2 were supported, our results provided no support for H3, H4, H5, H6, H7, and H8 in the ethos condition.

For the logos condition, communication strategy predicted trust. The results showed that the coefficient of communication strategy on trust was positive (*β*=.657; P<.001). The coefficient of trust on AI adoption was also positive and significant (*β*=.474; P=.008). The coefficients of communication strategy on perceived novelty value (*β*=.582; P<.001), customer innovativeness (*β*=.602; P<.001), and privacy concerns (*β*=−.267; P=.05) are significant . However, the coefficients of perceived novelty value (*β*=.104, P=.61), customer innovativeness (*β*=−.017; P=.91), and privacy concerns (*β*=−.074; P=.49) on AI adoption were insignificant, and the magnitude confirmed the previous theoretical findings that customer innovativeness and privacy concerns inhibited AI adoption. Although H5 and H6 were supported, our results did not support for H1, H2, H3, H4, H7, and H8 for logos.

## Discussion

### Overview

We empirically validated the influence of different communication strategies on overcoming factors that inhibit AI adoption. Having presented the results of our analysis (Table 3), we now consider the implications for users and research. We also discuss the limitations of this study and how they might inform future research initiatives.

Ethos-based communication strategies that rely on credibility and personal branding directly affect customer innovativeness, thereby increasing AI technology adoption. This can be because end users or patients can verify the veracity of these endorsers and discern and understand the credibility of ethos-based ads. By contrast, communication strategies based on logos and pathos independently aid in alleviating the trust issues that patients have, thereby increasing the adoption rate of AI. It is to be noted that trust has both emotional and logical parts. Hence, trust can be influenced emotionally (through pathos messaging) and logically (through logos messaging). Furthermore, a pathos-based communication strategy purposefully evokes emotions, thereby making end users feel the persuasion and connection to the product at a more personal level, leading them to identify the ease of use and usefulness of the AI product, which improves novelty value and AI adoption. Although pathos- and logos-based communication, which uses emotions and evidence to persuade, had a negligible impact on alleviating the privacy concerns of AI product users, questions remain about unethical data sharing and potential misuse of data by commercial organizations. This could be owing to information asymmetry between end users and organizations regarding how medical data may be used. Privacy concerns, including unethical data sharing and potential misuse of data by commercial organizations, continue to have a major impact on the adoption of AI technologies [45].

**Table 3:** Summary of the analysis.

Hypothesis	Pathos	Ethos	Logos
H1: using communication strategies for promoting the AI^a^ product can positively impact customer innovativeness.	Supported	Supported	Supported
H2: nudging customer innovativeness can improve acceptance of the AI product being promoted.	Not supported	Supported	Not supported
H3: using communication strategies for promoting the AI product can alleviate privacy concerns with using the product.	Supported	Not supported	Supported
H4: alleviating privacy concerns can improve acceptance of the AI product being promoted.	Not supported	Not supported	Not supported
H5: using communication strategies for promoting the AI product can improve trust in using the product.	Supported	Supported	Supported
H6: enhancing trust can lead to better acceptance of the AI product being promoted.	Supported	Not supported	Supported
H7: using communication strategies for promoting the AI product can positively influence the perceived novelty value of the product.	Supported	Supported	Supported
H8: perceived novelty value can lead to better acceptance of the AI product being promoted.	Supported	Not supported	Not supported

^a^AI: artificial intelligence.

Prior studies note that AI systems will not replace human clinicians on a large scale, but rather will augment their efforts to care for patients [21]. With AI being a new agent introduced into the care process, patients often need to share sensitive information with the system without being fully aware of the consequences of such actions. Through such actions, patients stand to gain in terms of temporal displacement of care (ie, using AI to displace later high-cost interventions in favor of earlier preventive procedures) [28]. Despite these benefits, patients risk the loss of privacy, face systemic inequality or discrimination because of embedded biases in the AI tool, and face the possibility of being subjected to errors or injuries because of miscalculations of the system. Using appropriate communication strategies can alleviate some of the concerns users may have about using a new agent in their care process.

Our findings make a key theoretical contribution to the technology adoption literature, specifically AI adoption in health care. Effective health communication with the public does not just happen, and this process of communicating with the public needs to be taught and practiced in health care [11]. Although it has been established that AI in health care is much more efficient than a clinician, the growth in the adoption of AI has been slower than expected because of various factors, such as the novelty of technology and other user concerns. Consumer research shows that people usually adhere to their existing routines, characterized by risk aversion [9], and that novelty value, along with emotional and social values, significantly influences the adoption of new technologies [17]. The AI adoption literature has examined the factors that can inhibit the adoption of a product. Although many medical AI products are marketed to their users, the role of communication strategies in overcoming inhibiting factors has received scant attention. This study addresses this research gap by identifying the underlying mechanism that each type of communication strategy can have on overcoming some of the inhibiting factors to improve AI adoption. It clearly notes users’ concerns regarding the adoption of AI technologies and which communication strategies work best to address these user concerns, thereby helping with quicker technology adoption.

### Limitations and Future Work

In the current rush toward using AI for aiding businesses in a variety of tasks [62] and with AI increasingly becoming integrated in many aspects of human life [63], we believe that communication strategies can help users transcend any perceived risks inherent to using AI products. Inducing pathos, ethos, and logos communication strategies improved AI adoption. In this study, we did not consider the effects of multiple communication strategies acting simultaneously on AI adoption; therefore, it would be beneficial for future studies to examine whether the effects of multiple communication strategies on AI adoption are additive. Another limitation of this study was that it did not consider the impact of communication strategies on AI adoption by various stakeholders (eg, health care practitioners, researchers, and patients). Health care practitioners are trained and possess more knowledge of the medical domain. Therefore, they may not be easily swayed through emotion-based communication strategies. Similarly, older users could be more apprehensive about privacy risks, thereby leading to less adoption among them [64,65]. Further research is required to investigate its effects on various stakeholders. In addition, we observed that the inhibiting factors were influenced differently by pathos, ethos, and logos communication strategies. For example, trust was influenced by pathos and logos but not by ethos. Thus, researchers can further examine the differential effects observed in our study for various communication strategies.

### Conclusions

In our research, we were able to establish that communication strategies influence the adoption of AI by effectively mitigating any concerns that end users might have regarding the adoption and use of medical AI products. The increased adoption of AI in the US health sector would be a major advantage from both efficiency and cost perspectives, resulting in improved patient well-being. Thus, although health AI would not fully replace human clinicians, increased adoption of AI aided by appropriate communication strategies would result in reduced cost and better affordability of health services by end users. Our research can be used by hospitals and clinicians for targeted ads and communication while trying to allay any user concerns related to AI in health care.

## Appendix

Multimedia Appendix 1 Literature review and experiment supplementary material.

## Abbreviations

AI: artificial intelligence
AMT: Amazon Mechanical Turk
H: hypothesis
PLS: partial least squares
TAM: Technology Acceptance Model
