# Supplementary material for: Persuading Patients Using Rhetoric to Improve Artificial Intelligence Adoption: Experimental Study
Source: J Med Internet Res. 2023 Mar 13;25:e41430. doi: 10.2196/41430 (PMC10131865; doi:10.2196/41430)
Supplement: Multimedia Appendix 1 [file jmir_v25i1e41430_app1.docx]

**Multimedia Appendix 1**

**Table S1: Overview of relevant literature**.

| **Topic** | **Study** | **Paper type, journal, year** | **Title** | **Findings and variables** | **Research gap** |
| --- | --- | --- | --- | --- | --- |
| Effective use of *communication strategies* | Green [18] | Review,  *Academy of Management Review, 2004* | A rhetorical theory of diffusion | Discusses the effective ways of using communication strategies. | Does not study the effect of rhetoric on adoption of emergent technologies (eg, AI^a^) in healthcare setting, where privacy concerns are high. In addition, regulations mandate protecting patient privacy. |
| Using *communication strategies* for health communication | Wieder [11] | Review,  *NCBI - National center for biotechnology information*, 2019 | Communicating radiation risk: the power of planned, persuasive messaging | Discusses a theoretical approach to using rhetoric (ethos, pathos, logos) to deliver a persuasive message on radiation risk. | The study is not empirical. Neither does it assess the influence of rhetoric on innovative technology product adoption in health care. |
| Role of *communication strategies* in influencing attitude on health data sharing | Sleigh and Vayena [19] | Experiment, *Humanities and Social Sciences Communications*, 2021 | Public engagement with health data governance: the role of visuality | Reason is not enough to motivate them to act; study concluded that rhetorical modes of logos (rational) and pathos (emotion) were used to change United Kingdom societal attitudes toward sharing health data. | While the study considered data sharing, it does not consider the influence of rhetoric (ethos, pathos, logos) on customer innovativeness and perceived novelty when adopting emerging technology (AI) product within health care. |
| Study on adoption of *communication strategies* in medical practice | Miles and Mezzich [20] | Review, *European Journal of Person-Centered Health Care*, 2014 | The care of the patient and the soul of the clinic: person-centered medicine as an emergent model of modern clinical practice | Study discusses on selecting Person-Centered Medicine (Pathos) over EBM^b^ and PCC^c^. Medical humanism called for physicians to return the profession to its caring roots, the pathos of medicine. | The study is not empirical. Further, it does not specifically measure the influence of rhetoric on innovative technology product adoption in health care. |
| Study on factors influencing *technology acceptance* | Davis [16] | Field & Lab study, *MIS Quarterly*, 1989 | Perceived usefulness, perceived ease of use, and user acceptance of information technology | Develops and validates new scales for 2 specific variables, perceived usefulness, perceived ease of use and user acceptance of IT. | Does not consider the influence of rhetoric on emergent technology (AI) product adoption in health care. |
| Convergence of human and *AI in medicine* | Topol [21] | Review Paper, *Nature Medicine*, 2019 | High-performance medicine: convergence of human and artificial intelligence | AI is much quicker and more accurate than clinicians.  Full automation with no potential for human backup of clinicians is not the objective. | Does not empirically measure the influence of rhetoric on emergent technology (AI) product adoption in health care. |
| *Resistance to AI* adoption in health care—Patients | Longoni et al [15] | Mix methods, *Journal of Consumer Research (Oxford University Press)*, 2019 | Resistance to medical artificial intelligence | Patient resistance to AI is due to uniqueness neglect, concern that AI providers are less able than human providers to account for user-unique characteristics. | While this study tries to understand the inhibitors in AI adoption, it does not measure the influence of rhetoric on AI adoption in the context of technology adoption model. |
| *User acceptance of information technology* | Venkatesh et al [22] | Longitudinal study, *MIS Quarterly*, 2003 | User acceptance of information technology: toward a unified view | The theory holds that there are four key constructs: (1) performance expectancy, (2) effort expectancy, (3) social influence, and (4) facilitating conditions. The first 3 are direct determinants of usage intention and behavior, and the fourth is a direct determinant of user behavior. | Does not consider and measure a facilitating condition of the influence of rhetoric on emergent technology (AI) product adoption in health care. |
| *Trust in AI* (based on a review of literature with focus on clinicians) | Asan et al [7] | Review, *Journal of Medical Internet Research*, 2020 | Artificial Intelligence and Human Trust in health care: focus on clinicians | Study supports the concept of optimal trust in which both humans and AI have some level of skepticism regarding the other’s decisions because both are capable of making mistakes. | Does not consider and measure the influence of rhetoric on overcoming trust barriers emotionally/logically in emergent technology (AI) product adoption in health care. |
| *Communication strategies* to persuade *Technology adoption* | Son and Han [10] | Experiment, *Journal of Business Research*, 2011 | Technology readiness effects on postadoption behavior | Concludes that communication strategies that the managers of technology-based products use to promote the usage of their technologies have an impact on technology adoption. | Does not measure the influence of rhetoric on user (patients) trust in the context of emergent technology (AI) product adoption in health care. |
| Consumer innovativeness and *technology adoption* | Baumeister [9] | Experiment, *Journal of Consumer Research*, 2004 | Self-control failure, impulsive purchasing, and consumer behavior | Consumer research shows that people are likely to stick with their existing routines, characterized by risk aversion and a general preference to buy familiar products. | Does not measure the influence of rhetoric on consumer (patient) innovativeness in the context of emergent technology (AI) product adoption in health care. |
| Impact of product Novelty on *technology adoption* | Hedman and Gimpel [17] | Interview/focus groups, *Information Technology and Management*, 2010 | The adoption of hyped technologies: a qualitative study | Research notes that novelty (epistemic) value, along with emotional and social values, significantly influence the adoption of new technologies. | Does not consider the influence of rhetoric on product novelty factor (for patient) in the context of emergent technology (AI) product adoption in health care. |
| Psychological antecedents that influence adoption of AI powered autonomous vehicles | Meyer-Waarden and Cloarec [14] | Experiment, *Technovation*, 2022 | “Baby, you can drive my car”: psychological antecedents that drive consumers’ adoption of AI products | Performance expectancy, user well-being and technology trust as well as user innovativeness influence the behavioral intention of user that in turn affects the technology adoption. | Does not measure the influence of rhetoric on psychological antecedents (of patients) in the context of emergent technology (AI) product adoption in health care. |
| Attributes influencing Consumers’ Adoption of Wearable health care Technology | Cheung et al [8] | Survey, *International Journal of Environmental Research and Public Health*, 2019 | Examining consumers’ adoption of wearable health care technology: the role of health attributes | Examines the influences of perceived usefulness, consumer innovativeness, and health information accuracy and privacy concerns on the adoption intention of wearable health care technology. | Does not measure the influence of rhetoric on technology adoption among patients in the context of emergent technology (AI) product adoption in health care. |

^a^AI: artificial intelligence.

^b^EBM: evidence-based medicine.

^c^PCC: patient-centered care movement.

**Table S2a: Experiment steps**

| 1. Randomly assign participants to various groups |
| --- |
| 2. Collect demographics (e.g., race, gender, country, etc.) |
| 3. Induce communication strategies (pathos, ethos, logos) * |
| • Logos: see ad in Figure 1  • Ethos: see ad in Figure 2  • Pathos: see ad in Figure 3 |
| 4. Manipulation check |
| 5. Measure trust, customer innovativeness, perceived novelty value, privacy concerns, and AI adoption |

* Primes given at end of appendix 1.

**Table S2b: Experiment Design**

| \|  \| **Communication strategy** \| \| \| \| \| --- \| --- \| --- \| --- \| --- \| \| **Pathos** \| **Logos** \| **Ethos** \| **Control** \| \| **Groups** \| **G1**  **(Figure 3 Ad)** \| **G2**  **(Figure 1 Ad)** \| **G3**  **(Figure 2 Ad)** \| **C1**  **(No Ad)** \| |
| --- | --- | --- | --- | --- | --- | --- | --- | --- | --- | --- | --- | --- | --- | --- |


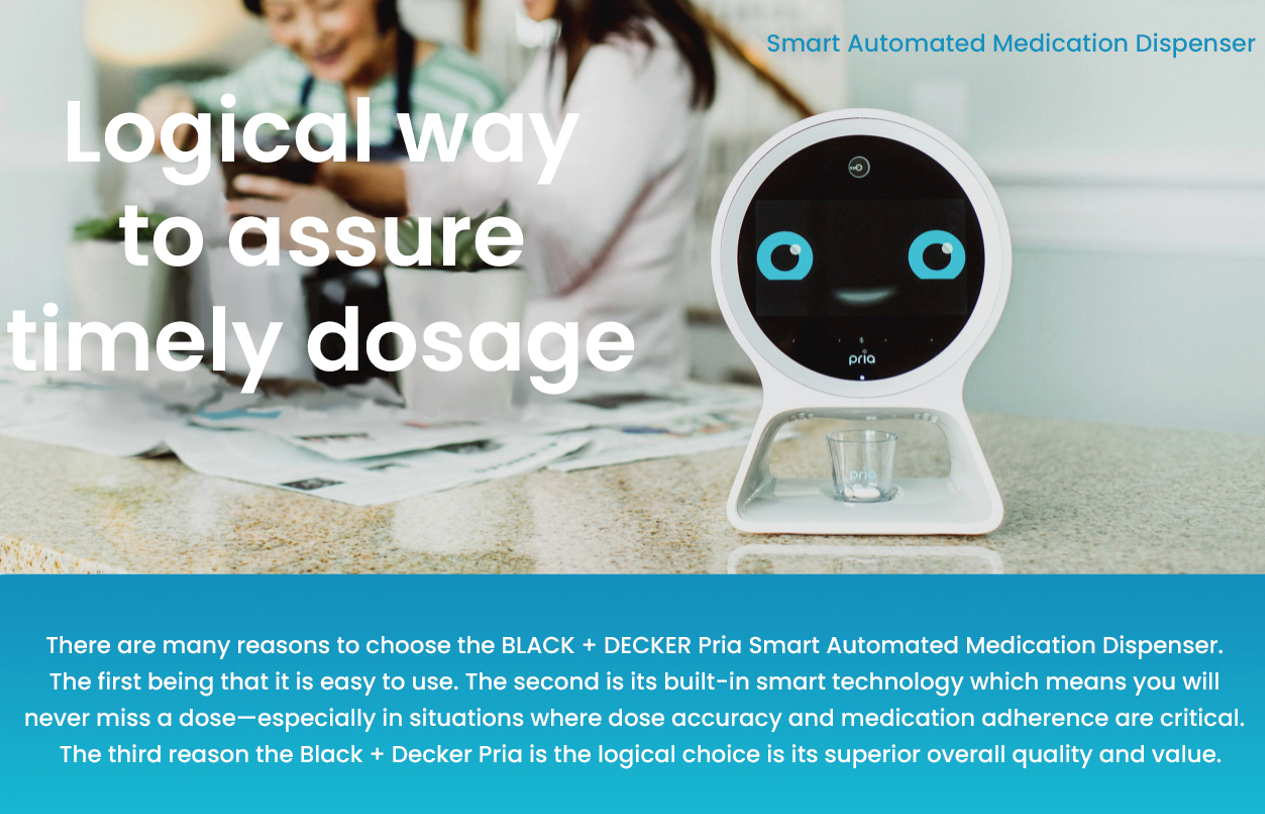


**Figure-1:** Ad1- Logos, persuasion using logical reasoning and evidence


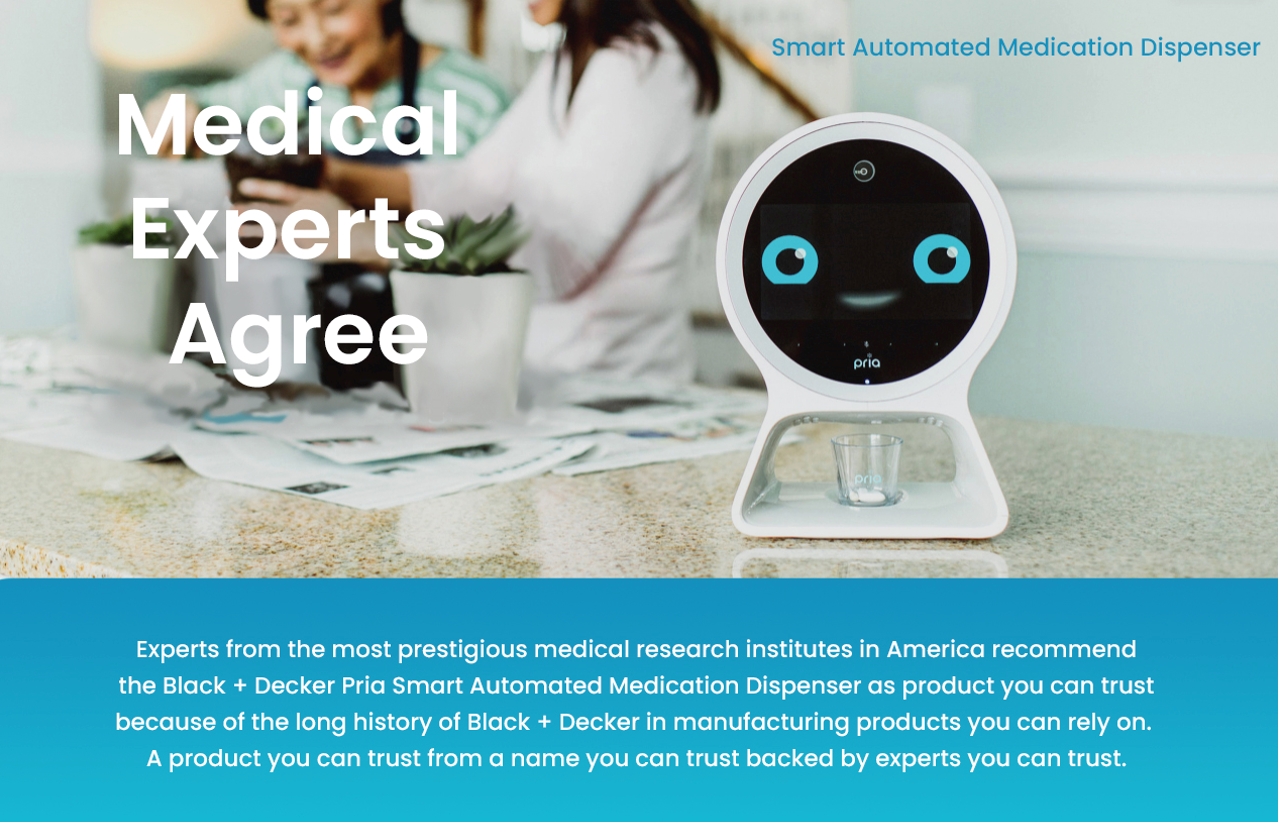


**Figure 2:** Ad2- Ethos, persuasion using credibility and ethics


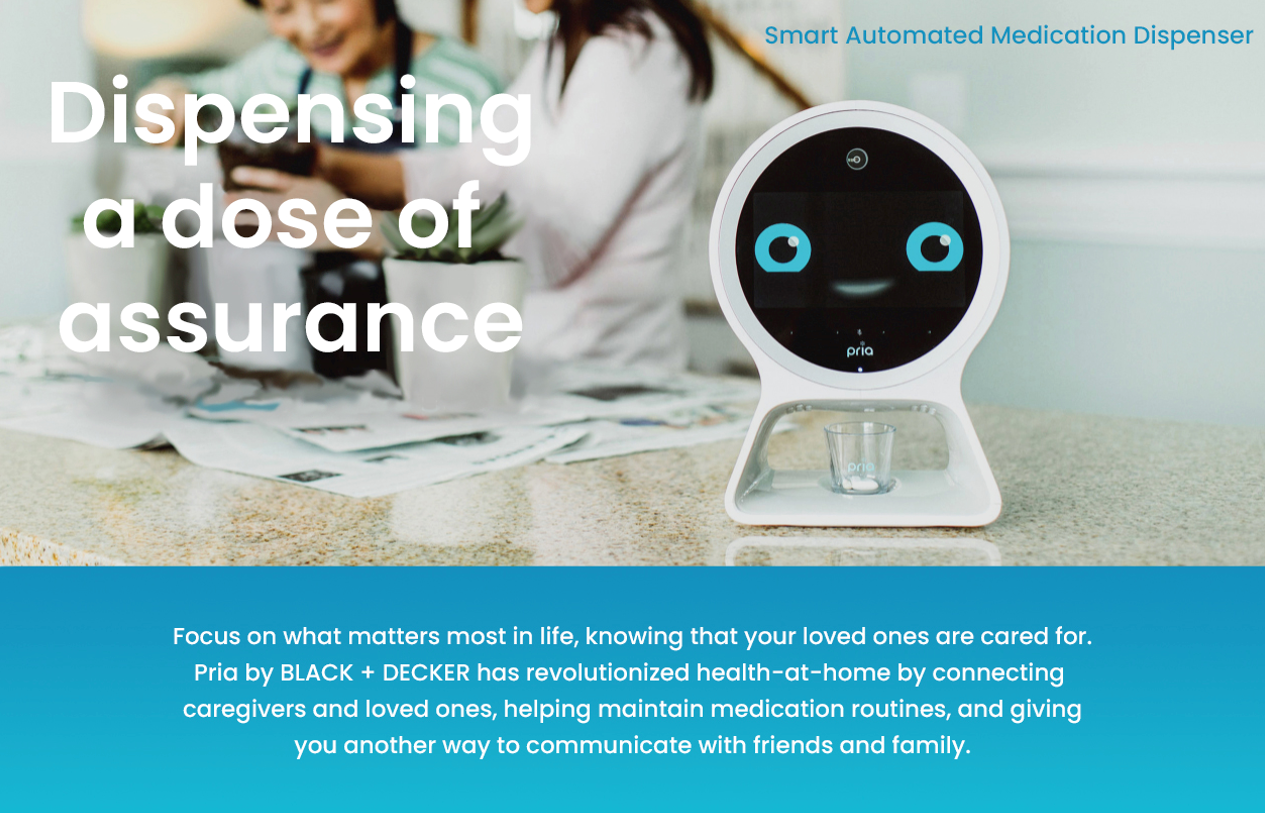


**Figure-3:** Ad3- Pathos, persuasion using emotion and passion

**Table S3: Survey Instrument**

| **Construct** | **Scale Items** |
| --- | --- |
| **AI Adoption** [56] | 1. AI will offer more consistent services than human beings  2. AI will offer more accurate services than human beings  3. AI will offer more efficient services than human beings  4. AI will offer cheaper services than human beings  5. AI will deliver a satisfactory experience  6. AI will enhance experience  7. AI will provide safe services  8. AI will be part of the healthcare landscape  9. AI will offer a modern look to healthcare  10. AI will replace jobs, where the employees’ livelihood is in danger  11. AI will replace repetitive jobs that do not need creativity  12. AI will replace doctors in the health system |
| **Privacy Concern (PC)** [55] | 1. I am concerned that the information about me could be misused.  2. I am concerned that others can get private information about me.  3. I am concerned about providing personal information, because of what others might do with it.  4. I am concerned about providing personal information, because it could be used in a way I cannot foresee. |
| **Trust** [52] | 1. I believe DaVinci surgical system acts in my best interest.  2. I expect DaVinci surgical system to be sincere and genuine.  3. I believe DaVinci surgical system performs its roles very well. |
| **Novelty Value** [53] | 1. Using DaVinci surgical system is a unique experience.  2. Using DaVinci surgical system is a once-in-a-lifetime experience.  3. Using DaVinci surgical system is an educational experience.  4. The experience of using DaVinci surgical system satisfies my curiosity.  5. Using DaVinci surgical system provides an authentic/genuine experience. |
| **Customer Innovativeness (CI)** [54] | 1. If I heard about new information technology, I would look for ways to experiment with it.  2. Among my peers,I am usually the first to explore new information technologies.  3. I like to experiment with new information technologies.  4. I get a kick out of using new high-tech services before most other people know they exist.  5. It is cool to be the first to own new high-tech services.  SD6. Being the first to use new high-tech services is very important to me. |

*Likert scale was used (1 Strongly agree … 7 Strongly disagree)

**Table S4: Manipulation check**

| **Priming Dimension** | **Manipulation check question** |
| --- | --- |
| Ad relevance, credibility, persuasiveness [6] | 1. This advertisement is relevant to me 2. This advertisement was meaningful to me 3. This advertisement is believable 4. This advertisement is important to me 5. This advertisement is realistic 6. This advertisement is convincing 7. This advertisement is persuasive 8. This advertisement is credible |
| Product involvement [39] | 1. This product is relevant to me 2. This product is irrelevant to me * 3. This product is important to me 4. This product category is unimportant to me * 5. This product means nothing to me 6. This product means a lot to me * |

* Reverse scale; Likert scale was used (1 Strongly agree … 5 Strongly disagree)

# Research Context:

Healthcare presents a context to examine the proposed research model because of various instantiations of AI products. Both structured and unstructured data are processed using machine learning, neural networks, modern deep learning, and natural language processing in medical AI applications. The most common application of AI is in precision medicine, i.e., predicting treatment protocols based on patient attributes and the treatment context (see Table S5). These tools are specifically used by oncologists and cardiologists. The AI tools use analytical algorithms to analyze patient health information (e.g., physical exam results, medications, symptoms, basic metrics, disease-specific data, diagnostic imaging, gene expressions, and different laboratory testing) to suggest treatments. AI technology thus is utilized in extracting truly relevant information from a large amount of medical data and applying it to diagnosis and treatment [7].

Healthcare has several tiers. The tier of healthcare that comes into contact with a patient, preventative health care, is the main responsibility of the primary health care sector. At the primary tier, the main AI tools are the AI Chatbots, which gather inputs from patients and provide medical advice and guidance. Patients with various abnormalities are referred to the secondary healthcare facilities. AI technologies used in the second tier include medical scanning, while the tertiary level includes robotic surgeries and drug delivery. Patients also use AI tools for achieving health-related goals. For example, AI tools are used in medication adherence. Patients use AI for reminders to take medication, and technology tools like Fitbit help users keep track of their daily steps. The list of healthcare tiers and overview of AI tools in healthcare is summarized in Table S5 below. As shown in Table S5, clinicians and patients have concerns about privacy, trust, innovativeness, and novelty that can influence their decision to adopt AI. Therefore, empirically validating the research hypotheses in the healthcare context is appropriate.

**Table S5: Overview of AI Tools in Healthcare and factors hindering/favoring adoption**

| **AI Tools** | **Adoption/acceptance** | **Factors hindering acceptance/adoption** | **Factors favoring acceptance/adoption** |
| --- | --- | --- | --- |
| AI products in **Primary healthcare** | Adoption is increased by efficiency and ease of use | **Patient:** privacy risk, lack of awareness, trust issues | Ease of use of chatbots |
| ML based tool - acts  as physician decision aid in **Secondary healthcare** | Becoming widely used due to the accuracy of the algorithm | **Clinician**: Supporting infrastructure, e.g., unstructured data from hospital systems, risk of workforce reduction, expected ROI, lack of trust for AI-based decisions | The proven effectiveness of the algorithm |
|  |  | **Patient**: Privacy risk, lack of awareness, lack of trust for AI-based decisions, uniqueness neglect | Effectiveness of algorithm |
| AI based robots  i. Surgeries  ii. Nanorobots for drug delivery  **Tertiary healthcare** | AI robots being used for  i. Surgery  ii. Nanorobots for drug delivery | **Clinician** : Risk of error by AI by lack of judgment, workforce upskilling to work with AI, ROI, firm size, lack of trust for AI-based decisions | Improved efficiency |
|  |  | **Patient**: Lack of awareness, risk of error/safety, cost, uniqueness neglect, lack of trust for AI-based decisions | Better precision |
| Automate administrator tasks, e.g., eligibility checks, insurance un-adjudicated claims, e.g., Olive AI | Not yet widely accepted, automating admin tasks can result in 30% cost savings, e.g., pre-authorizing insurance, following up on unpaid bills | **Clinician**: Digital maturity, firm size, expected ROI | Increased efficiency |
|  |  | **Patient**: Lack of awareness, lack of human interaction and care, uniqueness neglect | Increased efficiency |

**Table S6: Demographic information of participants**

|  | **Control** | **Ethos** | **Logos** | **Pathos** |
| --- | --- | --- | --- | --- |
| **Gender** |  |  |  |  |
| Female | 29 | 31 | 25 | 33 |
| Male | 17 | 17 | 19 | 17 |
| **Age** |  |  |  |  |
| 16-20 |  |  | 1 |  |
| 21-25 | 9 | 5 | 4 | 7 |
| 26-30 | 10 | 8 | 7 | 5 |
| 31-35 | 5 | 6 | 4 | 9 |
| 36-40 | 4 | 3 | 4 | 8 |
| 41-45 | 5 | 4 | 3 | 7 |
| 46-50 | 1 | 5 | 10 | 3 |
| 51-55 | 3 | 6 | 5 | 7 |
| 56-60 | 1 | 5 | 1 | 3 |
| Above 60 | 8 | 6 | 5 | 2 |
| **Race** |  |  |  |  |
| American Indian or Alaska Native | 1 | 1 | 1 | 1 |
| Asian | 3 | 3 | 5 | 2 |
| Black or African American | 7 | 6 | 3 | 6 |
| White | 35 | 35 | 33 | 38 |
| Multiracial | 2 | 1 | 2 | 2 |
| **Education** |  |  |  |  |
| Doctoral Degree | 2 | 2 |  | 2 |
| Graduate Degree | 14 | 8 | 8 | 12 |
| High School | 10 | 13 | 13 | 14 |
| Technical school or community College | 4 | 6 | 3 | 9 |
| Undergraduate Degree | 17 | 19 | 20 | 14 |

#### Appendix B

#### Measurement Model Assessment

Our main predictors are the communication strategy related dimensions. Each dimension was measured using a scale adapted from literature (see table S4). The trust, customer innovativeness, perceived novelty value, privacy concern and AI adoption variables were measured based on items adapted from prior literature (see Appendix 1, table S4) with a 5-point Likert scale and a mean score was computed. For constructs that were assessed using multiple items, exploratory factor analysis (EFA) with maximum likelihood extraction and varimax rotation was conducted to verify convergent and discriminant validity along with reliability tests.

We examined standardized loadings to assess the convergent validity of our reflective constructs. To ensure that the variance between each item and the associated construct exceeds the error variance, it is suggested that the standardized loadings (shown in Tables B1, B3, B5) should exceed 0.707 [58]. However, it is still acceptable for a measure to have a loading of 0.6 or higher if all other measures associated to the same construct have high loadings [58]. In order to assess the internal consistency of our measures for each construct, we examined Cronbach’s alpha, composite reliability, and average variance extracted for each construct. For Cronbach’s alpha and composite reliability, it is suggested that values of 0.7 or higher are adequate. All Cronbach’s *α* are well above the .70 threshold (see Table B2, B4, B6). With regard to AVE, the values should exceed 0.50 to ensure that more variance is captured by the measures relative to measurement error [22]. AVEs for constructs were above the recommended 0.5 threshold. AVE below 0.5 is acceptable if composite reliability is greater than 0.9. Given the assessment of convergent validity, all measures were retained for subsequent analysis.

To assess discriminant validity, we first examined the item loadings and cross-loadings on each construct. All measures had higher loadings for the intended construct than other constructs, providing there was evidence of discriminant validity (see Tables B1, B3, B5). Additionally, we calculated the squared correlation of all construct pairs and compared it with the AVE of each construct to ensure that more variance associated with each construct is captured by its indicators, rather than the indicators of other constructs [22]. The AVE for each construct exceeded the squared correlation of all construct pairs, thus providing further evidence of discriminant validity (see Table B2, B4, B6).

The results show strong support for convergent and discriminant validity. Based on the assessment of convergent and discriminant validity, we concluded that the measurement model was sufficiently robust to allow us to proceed to evaluation of the structural model.

#### Common Method Bias Analyses

Because trust, customer innovativeness, perceived novelty value, privacy concern and AI adoption were obtained using the same experimental instrument, we conducted a separate test to examine common method bias in our data. The test we conducted was Harmon’s single factor test [60], which involved an exploratory factor analysis with all items used to measure the main variables in our study. The communication strategy constructs (i.e., ethos, pathos, logos) are not susceptible to common method bias because they were experimentally manipulated in this study. Therefore, the communication strategy constructs were excluded from the tests of common method bias. The unrotated factor solution produced six factors with eigenvalues greater than 1, and with a total of 82.4% (ethos condition); 79.4% (pathos condition); and 81% (logos condition) of the variance accounted for. The first extracted factor accounted for 35.8% (ethos condition); 33.8% (pathos condition); and 35.6% (logos condition) of the variance in the data. These results suggest that common method bias is unlikely to be a significant problem in our data given that more than one factor emerged from the factor analysis, as well as the fact that the first factor did not account for the majority of the variance in our data.

**Communication Strategy Measure Validation (Pathos)**

***Table S7: Item loadings and cross loadings***

|  | **AIAdopt** | **CI** | **CS** | **Novelty** | **PC** | **Trust** |
| --- | --- | --- | --- | --- | --- | --- |
| **AIAdoptAVG** | **1.000** | 0.298 | 0.326 | 0.707 | -0.092 | 0.665 |
| **CI1** | 0.433 | **0.821** | 0.364 | 0.661 | -0.108 | 0.570 |
| **CI2** | 0.220 | **0.914** | 0.360 | 0.473 | -0.138 | 0.251 |
| **CI3** | 0.145 | **0.879** | 0.421 | 0.404 | -0.196 | 0.244 |
| **CI4** | 0.258 | **0.894** | 0.534 | 0.445 | -0.195 | 0.320 |
| **CI5** | 0.119 | **0.866** | 0.391 | 0.460 | -0.085 | 0.225 |
| **CI6** | 0.295 | **0.812** | 0.530 | 0.483 | -0.315 | 0.259 |
| **Novelty1** | 0.564 | 0.402 | 0.407 | **0.872** | -0.011 | 0.692 |
| **Novelty2** | 0.593 | 0.639 | 0.448 | **0.808** | -0.036 | 0.573 |
| **Novelty3** | 0.539 | 0.537 | 0.357 | **0.832** | 0.109 | 0.530 |
| **Novelty4** | 0.617 | 0.486 | 0.497 | **0.897** | -0.083 | 0.571 |
| **Novelty5** | 0.693 | 0.394 | 0.483 | **0.871** | -0.144 | 0.657 |
| **PC1** | -0.100 | -0.174 | -0.303 | -0.063 | **0.943** | -0.098 |
| **PC2** | -0.111 | -0.203 | -0.317 | -0.053 | **0.978** | -0.073 |
| **PC3** | -0.049 | -0.211 | -0.309 | -0.016 | **0.975** | -0.040 |
| **PC4** | -0.092 | -0.225 | -0.351 | -0.050 | **0.976** | -0.071 |
| **Trust1** | 0.635 | 0.273 | 0.487 | 0.604 | -0.065 | **0.937** |
| **Trust2** | 0.667 | 0.421 | 0.592 | 0.724 | -0.135 | **0.935** |
| **Trust3** | 0.504 | 0.312 | 0.347 | 0.610 | 0.029 | **0.880** |

***Table S8: Correlations, AVE, and Cronbach alpha***

|  | **AIAdopt** | **CI** | **CS** | **Novelty** | **PC** | **Trust** |
| --- | --- | --- | --- | --- | --- | --- |
| **AIAdopt** | 1.000 |  |  |  |  |  |
| **CI** | 0.298 | 0.865 |  |  |  |  |
| **CS** | 0.326 | 0.514 | 1.000 |  |  |  |
| **Novelty** | 0.707 | 0.570 | 0.517 | 0.857 |  |  |
| **PC** | -0.092 | -0.211 | -0.332 | -0.047 | 0.968 |  |
| **Trust** | 0.665 | 0.370 | 0.532 | 0.707 | -0.073 | 0.918 |

|  | **Cronbach's Alpha** | **Composite Reliability** | **Average Variance Extracted (AVE)** |
| --- | --- | --- | --- |
| **AIAdopt** | 1.000 | 1.000 | 1.000 |
| **CI** | 0.933 | 0.947 | 0.748 |
| **CS** | 1.000 | 1.000 | 1.000 |
| **Novelty** | 0.909 | 0.932 | 0.734 |
| **PC** | 0.977 | 0.983 | 0.937 |
| **Trust** | 0.908 | 0.941 | 0.842 |

**Communication Strategy Measure Validation (Ethos)**

***Table S9: Item loadings and cross loadings***

|  | **AIAdopt** | **CI** | **Novelty** | **PC** | **Trust** |
| --- | --- | --- | --- | --- | --- |
| **AIAdopt** | **1.000** | **0.**646 | 0.638 | 0.060 | 0.617 |
| **Cust_Innovativeness1** | 0.654 | **0.777** | 0.370 | -0.123 | 0.321 |
| **Cust_Innovativeness2** | 0.529 | **0.893** | 0.408 | -0.201 | 0.406 |
| **Cust_Innovativeness3** | 0.541 | **0.839** | 0.295 | -0.159 | 0.308 |
| **Cust_Innovativeness4** | 0.450 | **0.883** | 0.376 | -0.132 | 0.441 |
| **Cust_Innovativeness5** | 0.607 | **0.934** | 0.402 | -0.158 | 0.428 |
| **Cust_Innovativeness6** | 0.449 | **0.743** | 0.448 | -0.138 | 0.489 |
| **Novelty1** | 0.430 | 0.333 | **0.792** | 0.070 | 0.597 |
| **Novelty2** | 0.416 | 0.358 | **0.662** | 0.014 | 0.490 |
| **Novelty3** | 0.567 | 0.345 | **0.807** | 0.131 | 0.640 |
| **Novelty4** | 0.489 | 0.316 | **0.878** | 0.059 | 0.682 |
| **Novelty5** | 0.612 | 0.457 | **0.835** | 0.032 | 0.688 |
| **PC1** | -0.010 | -0.273 | -0.035 | **0.953** | -0.128 |
| **PC2** | 0.043 | -0.219 | 0.059 | **0.958** | -0.075 |
| **PC3** | 0.093 | -0.135 | 0.105 | **0.983** | -0.013 |
| **PC4** | 0.062 | -0.145 | 0.110 | **0.973** | -0.029 |
| **Trust1** | 0.485 | 0.505 | 0.701 | -0.100 | **0.883** |
| **Trust2** | 0.453 | 0.336 | 0.627 | -0.143 | **0.885** |
| **Trust3** | 0.654 | 0.360 | 0.698 | 0.107 | **0.829** |

***Table S10: Correlations, AVE, and Cronbach alpha***

|  | **AIAdopt** | **CI** | **CS** | **Novelty** | **PC** | **Trust** |
| --- | --- | --- | --- | --- | --- | --- |
| **AIAdopt** | 1.000 |  |  |  |  |  |
| **CI** | 0.646 | 0.847 |  |  |  |  |
| **CS** | 0.629 | 0.465 | 1.000 |  |  |  |
| **Novelty** | 0.638 | 0.452 | 0.666 | 0.798 |  |  |
| **PC** | 0.060 | -0.180 | 0.106 | 0.079 | 0.967 |  |
| **Trust** | 0.617 | 0.466 | 0.662 | 0.783 | -0.048 | 0.866 |

|  | **Cronbach's Alpha** | **Composite Reliability** | **Average Variance Extracted (AVE)** |
| --- | --- | --- | --- |
| **AIAdopt** | 1.000 | 1.000 | 1.000 |
| **CI** | 0.920 | 0.938 | 0.718 |
| **CS** | 1.000 | 1.000 | 1.000 |
| **Novetly** | 0.857 | 0.897 | 0.637 |
| **PC** | 0.978 | 0.983 | 0.935 |
| **Trust** | 0.833 | 0.900 | 0.750 |

**Communication Strategy Measure Validation (Logos)**

***Table S11: Item loadings and cross loadings***

|  | **AIAdopt** | **CI** | **Novelty** | **PC** | **Trust** |
| --- | --- | --- | --- | --- | --- |
| **AIAdopt** | **1.000** | 0.406 | 0.578 | -0.330 | 0.715 |
| **Cust_Innovativeness1** | 0.385 | **0.843** | 0.544 | -0.162 | 0.358 |
| **Cust_Innovativeness2** | 0.284 | **0.906** | 0.370 | -0.192 | 0.279 |
| **Cust_Innovativeness3** | 0.487 | **0.846** | 0.573 | -0.196 | 0.559 |
| **Cust_Innovativeness4** | 0.320 | **0.850** | 0.513 | -0.047 | 0.427 |
| **Cust_Innovativeness5** | 0.246 | **0.742** | 0.324 | -0.103 | 0.363 |
| **Cust_Innovativeness6** | 0.219 | **0.801** | 0.335 | -0.137 | 0.210 |
| **Novelty1** | 0.577 | 0.335 | **0.758** | -0.104 | 0.634 |
| **Novelty2** | 0.288 | 0.422 | **0.638** | 0.038 | 0.319 |
| **Novelty3** | 0.287 | 0.394 | **0.665** | 0.075 | 0.283 |
| **Novelty4** | 0.463 | 0.576 | **0.897** | -0.317 | 0.606 |
| **Novelty5** | 0.481 | 0.360 | **0.772** | -0.464 | 0.643 |
| **PC1** | -0.362 | -0.127 | -0.262 | **0.945** | -0.329 |
| **PC2** | -0.283 | -0.138 | -0.247 | **0.923** | -0.255 |
| **PC3** | -0.326 | -0.246 | -0.323 | **0.934** | -0.408 |
| **PC4** | -0.211 | -0.102 | -0.096 | **0.892** | -0.329 |
| **Trust1** | 0.661 | 0.463 | 0.634 | -0.320 | **0.940** |
| **Trust2** | 0.659 | 0.478 | 0.580 | -0.328 | **0.944** |
| **Trust3** | 0.677 | 0.338 | 0.732 | -0.357 | **0.912** |

***Table S12: Correlations, AVE, and Cronbach alpha***

|  | **AIAdopt** | **CI** | **CS** | **Novelty** | **PC** | **Trust** |
| --- | --- | --- | --- | --- | --- | --- |
| **AIAdopt** | 1.000 |  |  |  |  |  |
| **CI** | 0.406 | 0.833 |  |  |  |  |
| **CS** | 0.609 | 0.602 | 1.000 |  |  |  |
| **Novelty** | 0.578 | 0.550 | 0.582 | 0.752 |  |  |
| **PC** | -0.330 | -0.172 | -0.267 | -0.266 | 0.924 |  |
| **Trust** | 0.715 | 0.456 | 0.657 | 0.699 | -0.360 | 0.932 |

|  | **Cronbach's Alpha** | **Composite Reliability** | **Average Variance Extracted (AVE)** |
| --- | --- | --- | --- |
| AIAdopt | 1.000 | 1.000 | 1.000 |
| CI | 0.912 | 0.931 | 0.694 |
| CS | 1.000 | 1.000 | 1.000 |
| Novelty | 0.810 | 0.865 | 0.565 |
| PC | 0.943 | 0.959 | 0.853 |
| Trust | 0.924 | 0.952 | 0.869 |
